# Outcomes of stroke patients undergoing thrombolysis in Sri Lanka; an observational prospective study from a low-middle income country

**DOI:** 10.1186/s12883-021-02475-3

**Published:** 2021-11-09

**Authors:** H. M. M. T. B. Herath, Chaturaka Rodrigo, A. M. B. D. Alahakoon, Sathyajith Buddhika Ambawatte, Sunethra Senanayake, Bimsara Senanayake, Arjuna Fernando

**Affiliations:** 1 Neurology Department, National Hospital of Sri Lanka, Colombo, Sri Lanka; 2 Department of Pathology, School of Medical Sciences, UNSW Sydney, Sydney, NSW Australia

**Keywords:** Stroke, Thrombolysis, Sri Lanka, Observational prospective study

## Abstract

**Background:**

Stroke related deaths are relatively higher in low- and middle-income countries where only a fraction of eligible patients undergo thrombolysis. There is also limited evidence on post-thrombolysis outcomes of patients from Asian countries in these income bands.

**Methods:**

This is a single center prospective observational study of a patient cohort with acute ischaemic stroke, undergoing thrombolysis with alteplase (low and standard dose), over a 24-month period in 2019/2020. Modified Rankin scale (mRS) for dependency at 3 months (primary outcome), duration of hospital stay, incidence of symptomatic intracranial haemorrhages and all-cause mortality at 3 months (secondary outcomes) were recorded. Demographic, clinical and treatment related factors associated with these outcomes were explored.

**Results:**

Eighty-nine patients (males – 61, 69%, mean age: 60 years ±12.18) were recruited. Time from symptom onset to reperfusion was 174 min ± 56.50. Fifty-one patients were independent according to mRS, 11 (12.4%) patients died, and 11 (12.5%) developed symptomatic intracranial haemorrhages by 3 months. Functional independence at 3 months was independently associated with National Institutes of Health Stroke Scale (NIHSS) on admission (*p* < 0.05). Thrombolysis with low dose alteplase did not lead to better or worse outcomes compared to standard dose.

**Conclusions:**

On admission NIHSS is predictive of functional independence at 3 months post-thrombolysis. Low dose alteplase may be as efficacious as standard dose alteplase with associated cost savings, but this needs to be confirmed by a prospective clinical trial for the Sri Lankan population.

**Supplementary Information:**

The online version contains supplementary material available at 10.1186/s12883-021-02475-3.

## Background

Globally, age standardized stroke mortality has declined from 2005 to 2017, but most of these deaths still occur in middle – and low- income countries [1]. Interestingly, age standardized stroke prevalence has been increasing since 2005 in middle income countries. Age standardized stroke related disability adjusted life years lost (DALYs) has been declining in countries across all income bands, probably reflecting advances in stroke care and therapy in the last 15 years, but there is still a huge discrepancy between high – income countries and others [1]. Overall, these numbers reflect the global inequality in resource distribution leading to suboptimal care and more deaths in stroke patients from less affluent countries. Thrombolysis with intravenous alteplase (a recombinant tissue plasminogen activator – rtPA) is one of the most important therapies in acute stroke management, but remains out of reach for many eligible candidates in lower – middle income countries (as low as 2% of stroke patients receive thrombolysis according to some studies) [2, 3], partly due to unavailability of the drug, public unawareness, and also due to lack of infrastructure (roads and transportation, paramedic services) which prevents patients reaching hospitals within the specified time window [2]. In addition, most of these countries have no locally developed guidelines to manage acute stroke patients, have fewer hospitals with dedicated stroke units, and have less accessible brain imaging when adjusted for the sizes of their populations [4]. Clinical research data on thrombolysis for ischaemic stroke from low-middle- income countries are also limited, compared to high income countries [5].

Sri Lanka is a low-middle income country with a population of approximately 22 million, with a dual public and private health care system. The public healthcare system is free for all citizens (funded by taxpayer) but accessibility is a problem due to high demand and overcrowding. By 2018, there were only 38 neurologists in the public health system, thrombolysis with rtPA was available in only 14 state hospitals and mechanical thrombectomy was available in only one hospital [6]. Thrombolysis is available in several private hospitals in a few main cities, and a private hospital in Colombo has been providing endovascular treatment since 2013. Thrombolysis with rtPA first started in Sri Lanka in 2008 but less people received this service as it was limited to neurology units in the National Hospital of Sri Lanka (NHSL-C), located in the commercial capital of Colombo. Also, most people could not get into the hospital in time due to lack of a paramedic and ambulance service and the distance from hospital, further complicated by traffic congestion. Since then, this service have expanded to more centers outside of Colombo, more trained neurologists joined the workforce and an island-wide paramedic and ambulance service had started since 2016 [7]. However, there is no data from Sri Lanka on the success of rtPA therapy, its complications and functional improvement observed on follow up.

The primary aim of this study is to describe the outcomes (functional independence at 3 months post-thrombolysis, duration of hospital stay, complications of symptomatic intracranial haemorrhage and death) of a cohort of patients that underwent thrombolytic therapy at the National Hospital of Sri Lanka over a 24-month period. The secondary aim was to identify demographic, clinical, and treatment (low vs. standard dose alteplase) related factors associated with a better outcome.

## Methods

This is an observational prospective study carried out at the National Hospital of Sri Lanka in Colombo (NHSL-C) between 2019 January - 2020 December. The study was reported according to STROBE guideline and extension. This hospital, located in the commercial capital and the most densely populated district of Sri Lanka, is the country’s largest and premier public health hospital. NHSL-C has three neurology units offering thrombolytic therapy and all of them were involved in this study. All adult consenting patients (> 18 years of age) with acute ischmeic stroke who were eligible for thrombolytic therapy according to American Heart Association/American Stroke Association (AHA/ASA) guidelines (2019 update) were included in the study if they had no contraindications for thrombolysis [8].

Of the three Neurology units at NHSL-C, one unit used low dose alteplase (0.7 mg/kg of actual body weight, with a maximum dose of 90 mg) as per pre-existing unit policy at the time of the study. Other two units used alteplase 0.9 mg/kg of actual body weight (maximum dose - 90 mg, henceforth referred to as “standard” dose). Hence the dose of alteplase received by a patient was determined by where the patient was admitted to and not as part of this study (the patient would have received the same treatment regardless of recruitment to this study). However, this provided an opportunity to observe if low dose alteplase was as effective as standard dose alteplase. The demographic (e.g., age, gender), clinical (past medical history, details of current presentation including time elapsed since onset of symptoms to admission or reperfusion), biochemical, haematological and imaging (non-contrast CT of head prior to thrombolysis) data for were recorded for all patients, before and after thrombolysis. The definitions used for each data item are further explained in supplementary Table 1. When a laboratory investigation was repeated, the earliest available results after the onset of symptoms was recorded. The National Institutes of Health Stroke Score (NIHSS) was calculated on admission prior to thrombolysis. The Alberta Stroke Programme early CT score (ASPECTS) was calculated retrospectively using the pre-thrombolysis non-contrast CT scan of the head [9]. After thrombolysis, all patients were monitored in a high dependency unit at least for 24 h with strict blood pressure control. Anticoagulant plus antithrombotic agents as well as invasive procedures (intra-arterial catheters, nasogastric tube insertion) were avoided as much as possible within the first 24 h post-thrombolysis. If a patient developed sudden neurologic deterioration, a decline in level of consciousness, new onset headache, nausea and vomiting, or a sudden rise in blood pressure post-thrombolysis, non-contrast CT head was repeated urgently. Otherwise, a repeat CT scan was done 24 h post-thrombolysis for all patients.

The primary outcome of interest was the modified Rankin Scale score (mRS) at 3 months (dichotomized as independent and dependent: 0-2 or ≥ 3, respectively )[10, 11]. Secondary outcomes were mRS at 3 months dichotomized as favourable (0-1) or unfavourable (≥ 2), duration of hospital stay, all-cause mortality at 3 months, and occurrence of a symptomatic intracranial haemorrhage (sICH) as defined by the National Institute of Neurological Disorders and Stroke (NINDS) criteria.

Data were analysed with Statistical Package of Social Science (SPSS version 21, IBM, USA). Descriptive statistics were summarized as measures of central tendency (mean / median) and dispersion (standard deviation or interquartile range) according to normality of distributions. Statistically significant differences were explored with independent T test or linear regression for continuous variables and with chi square test for categorical variables (unadjusted analysis). Any variables found to be significant in the unadjusted analysis were further explored with logistic regression (adjusted analysis). Cut-off for statistical significance was *p* < 0.05.

## Results

Eighty-nine patients with ischaemic stroke, who were eligible for rtPA were recruited (mean age: 60 years ±12.2, males = 61, 69%). The demographic characteristics, clinical characteristics on admission, and outcomes assessed 3 months after thrombolysis are summarised in Tables 1, 2, 3 and 4. Time from onset of symptom onset to admission, time from admission to the CT scan and time from symptom onset to reperfusion were 115 ± 58.3, 34 ± 33.3 and 174 ± 56.5 respectively (in minutes) for the entire cohort. Thirty-seven patients (43%) were reperfused within 3 h while the remainder were reperfused between 3 and 4.5 h since the onset of symptoms. The average NIHSS was 13 ± 5.2 on admission. Eighteen patients (18/89, 20.2%) received low dose rtPA while the remainder received the standard dose.

**Table 1:** Clinical and demographic characteristics of eligible patients

Characteristic	Mean ± SD	Category	Number (%)
Age (years)	60 ± 12.2	Less than 45	13 (14.6)
		45-55	11 (12.4)
		55-65	26 (29.2)
		65- 75	33 (37.1)
		More than 75	6 (6.7)
Gender	NA	Male	61 (68.5)
		Female	28 (31.5)
BMI	24 ± 3.9	Less than 18	1 (1.12)
		18 – 23	36 (40.4)
		23 – 30	46 (51.6)
		30 -35	5 (5.6)
		More than 35	1(1.12)
Cardiovascular comorbidities^a^	NA	No	15 (16.9)
		Yes	74 (83.1)
Smoking	NA	Non- or former smoker	69 (77.5)
		Current smoker	20 (22.5)
Alcohol Use (within last year)	NA	Non-user or < 90 g per week	77 (86.5)
		> 90 g per week	12 (13.5)
Aspirin use (regularly for last 2 weeks)	NA	No	49 (55.1)
		Yes	40 (44.9)
Clopidogrel use (regularly for last 2 weeks)		No	66 (74.2)
		Yes	23 (25.8)
Anticoagulant use	NA	No	84 (94.4)
		Yes	5 (5.6)
Statins use	NA	No	37 (41.6)
		Yes	52 (58.4)

^a^Includes diabetes mellitus (*n* = 44), hypertension (*n* = 57), previous stroke (*n* = 25), ischaemic heart disease (*n* = 22), heart failure (*n* = 15), atrial fibrillation (*n* = 13)

**Table 2:** Investigation and assessment scores on admission (pre-thrombolysis)

Characteristic	Mean ± SD	Category	Number (%)
Serum glucose on admission (mg / dL)	166 ± 69.7	50 – 200	66 (74.2)
		200 -350	19 (21.3)
		> 350	4 (4.5)
Systolic blood pressure on admission (mm/Hg)	162 ± 26.4	< 90	0 (0.0)
		90 – 140	19 (21.3)
		140 – 160	27 (30.3)
		160 – 185	26 (29.2)
		> 185	17 (19.1)
Diastolic blood pressure on admission (mm/Hg)	96 ± 14.5	< 60	0 (0.0)
		60 – 80	10 (11.4)
		80 – 90	27 (30.7)
		> 90	51 (58.0)
On admission mRS	04 ± 1	1	3 (3.4)
		2	11 (12.5)
		3	20 (22.7)
		4	46 (52.3)
		More than Four	8 (9.0)
On admission NIHSS	13 ± 5.2	0 – 4	4 (4.7)
		4 – 8	13 (15.1)
		8 – 12	18 (20.9)
		16 – 20	27 (31.4)
		20 – 24	18 (20.9)
		24 – 28	6 (7.0)
		> 28	0 (0.0)
On admission CT scan ASPECTS	9 ± 0.88		
On admission CT scan leukoarioisis	NA	Present	29 (32.6)
		Not present	60 (67.4)
On admission CT scan hyperdense cerebral artery sign	NA	Present	12 (13.5)
		Not present	77 (86.5)
Platelet count on admission	NA	. < 150,000	6 (6.7)
		150,000- 300,000	65 (73.0)
		> 300,000	18 (20.2)
On anticoagulant/s with INR ≤1.7	NA	Yes	5 (5.6)
		No	84 (94.4)
eGFR on admission	NA	> 90	41 (41.6)
		60 – 90	39 (44.3)
		30- 60	5 (5.7)
		15 – 30	3 (3.4)
		< 15	0(0.0)
Haematocrit level on admission	NA	Low	10 (11.2)
		Normal	73 (82.0)
		High	6 (6.7)
Stroke vascular territory	NA	Anterior	82 (92.1)
		Posterior	7 (7.9)

*mRS* modified Rankin scale, *NIHSS* National Institutes of Health Stroke Score, *ASPECTS* Alberta Stroke Programme early CT score, *INR* International normalized ratio, *eGFR* estimated glomerular filtration rate. Haematocrit level = The normal haematocrit for men is 40 to 54%; for women it is 36 to 48%

**Table 3:** Post-thrombolysis functional assessment

Characteristic	Mean ± SD	Category	Number (%)
Duration of hospital stay (days)	10 ± 7.7	NA	NA
mRS at 3 months		Independent (0 – 2)	51 (63.8)
		Dependent (3-5)	29 (36.2)
mRS at 3 months		Favourable (0 – 1)	37 (46.3)
		Unfavorable (2-5)	43 (53.8)

*mRS* modified Rankin scale, *NA* Not applicable

**Table 4:** Post thrombolysis complications

Characteristic	Category	Number (%)
Death at 3 months	No	79 (88.7)
	Yes	10 (11.3)
Cause of death at 3 months	Death due to ICH	7(63.6%)
	Death due to other causes	3(27.3%)
	Indeterminate	1 (9.1%)
Symptomatic ICH	No	80 (89.9)
	Yes	9 (10.1)

*ICH* Intracranial haemorrhage defined according to NINDS criteria

Regarding the primary outcome, 51 patients were categorized as independent by the mRS (score of 0 – 2) at 3 months (51/80, 63.7%). For secondary outcomes, 37 (37/80, 46.3%) patients had a favourable mRS score (0 – 1) by 3 months, 11 (11/89, 12.4%) patients developed sICH, and 11 (11/88, 12.5%) patients died within 3 months of thrombolysis. The median duration of hospital stay was 10 ± 7.7 days. Of the deaths, 7 were due to intracranial haemorrhages, 2 due to pneumonia and 1 due to cardiac cause. The cause was indeterminate for the other. In addition, 13 (14.9%) patients developed mild systemic bleeding following thrombolysis and none developed orolingual angioedema.

In the unadjusted analysis as shown in Table 5, several variables had a statistically significant association with the primary and secondary outcomes (*p* < 0.05). In the adjusted analysis, dependence at 3 months as assessed by mRS was independently associated with NIHSS on admission (aOR: 1.26, 95% CI: 1.09 – 1.44) only. Regarding secondary outcomes, an unfavourable mRS was independently associated with the NIHSS on admission (aOR: 1.22, 95% CI: 1.07 – 1.38), time to reperfusion (aOR: 1.02, 95% CI: 1– 1.04), and leukoaraiosis on pre-thrombolysis CT scan (aOR: 4.32, 95% CI: 1.08 – 17.22). There were no significant associations for the duration of hospital stay, occurrence of sICH or death in the adjusted analysis.

**Table 5:** Associations for functional outcomes, duration of hospital stay and complications at 3 months post-thrombolysis

Variable	*P* value against each outcome				
	*Independent mRS at 3 months*	*Favourable mRS at 3 months*	*Duration of hospital stay*	*Intracranial Haemorrhage at 3 months*	*Death at 3 months*
*History*					
Age^a^	NS	NS	NS	NS	NS
BMI^a^	NS	NS	NS	NS	< 0.001
Time to admission^a^	0.012	0.026	NS	NS	NS
Time to reperfusion^a^	NS	0.009^b^	NS	NS	NS
Diabetes mellitus	NS	0.044	NS	NS	NS
Hypertension	NS	NS	NS	NS	NS
Previous ischaemic heart disease	NS	NS	NS	NS	NS
Atrial fibrillation	NS	NS	NS	NS	NS
Heart failure	NS	NS	NS	NS	NS
Previous stroke	NS	NS	NS	NS	NS
Smoking	NS	NS	NS	NS	NS
On aspirin	NS	NS	NS	NS	NS
On clopidogrel	NS	NS	NS	NS	NS
On statins	NS	NS	NS	NS	NS
*Examination and investigations*					
Vascular territory of stroke	NS	NS	NS	NS	NS
On admission NIHSS^a^	< 0.001^b^	0.001^b^	0.044	NS	NS
CT- ASPECT score^a^	NS	NS	NS	NS	NS
CT – MCA hyperdense sign	NS	NS	NS	NS	0.040
CT - Leukoaraiosis	0.002	< 0.001^b^	NS	NS	NS
Glucose^a^	NS	NS	NS	NS	NS
SBP ^a^	NS	0.04	NS	NS	NS
DBP ^a^	NS	0.009	0.017	NS	NS
Platelet count	NS	NS	NS	NS	NS
INR < 1.7	NS	NS	NS	NS	NS
eGFR	NS	NS	NS	NS	NS
Haematocrit	NS	NS	0.019	NS	NS
*Treatment*					
Low vs. high dose rTPA	0.031	NS	NS	NS	NS

^a^continuous variables – evaluated with independent T test, All other categorical variables were evaluated with chi square test, NS – non-significant, ^b^ associations that remained significant in the adjusted analysis, *BMI* body mass index, *SBP* Systolic blood pressure, *DBP* Diastolic blood pressure, *CT* Computed tomography, *rTPA* recombinant tissue plasminogen inhibitor, *NIHSS* National Institutes of Health Stroke Score, *mRS* modified Rankin Scale, *eGFR* estimated glomerular filtration rate, *MCA* middle cerebral artery, *INR* international normalised ratio, *ASPECTS* Alberta Stroke Programme early CT score, *NS* Not significant

Though low dose rtPA was statistically significantly associated with the primary outcome in the unadjusted analysis (*p* = 0.031), this difference disappeared in the adjusted analysis. There was also no difference between low and standard dose rtPA treatment with regard to deaths, incidence of intracranial haemorrhages (or any other episodes of major or minor bleeds), or the duration of hospital stay.

## Discussion

This prospective observational study of patients with acute ischaemic stroke who were eligible for thrombolysis in a resource limited setting in Sri Lanka showed that nearly 12% of patients developed sICH or died within 3 months of thrombolysis. Of the survivors, more than 60% were functionally independent at 3 months and this was predicted by the on admission NIHSS. There was no difference in outcomes depending on whether low or standard dose rtPA was administered for thrombolysis.

The age distribution and NIHSS on admission in the patient cohort of this study were comparable to those observed in other prospective studies on thrombolysis for stroke as detailed in supplementary Table 2. However, the time from symptom onset to thrombolysis was comparatively higher (Supplementary Fig. 1). This may be due to delays in getting to hospital due to not using paramedic services, traffic congestion, living further away from the hospital or a combination of this factors. This study did not explore the reasons for delay in hospital admission, and also did not capture the people who came after the 4.5-h time window to compare with, but this highlights the need for more qualitative and quantitative data to explore the reasons for delays in getting to hospital. Earlier thrombolytic treatment reduces in-hospital mortality and symptomatic intracranial haemorrhages while increasing functional independence following treatment [12], and it’s important that patients in resource limited settings can tap into these benefits by timely access to healthcare.

Functional independence at 3 months post-thrombolysis in our patient cohort was associated with the on-admission NIHSS. Furthermore a “favourable” score of the mRS (0-1) was also associated with the on admission NIHSS, time to reperfusion and presence of leukoaraiosis on the pre-thrombolysis CT brain imaging. These observations agree with that of several previous studies [13–18]. However, other studies have also found different associations for functional dependence/independence at 3 months such as time to admission, major neurological improvement at 24 h [13], pre-thrombolysis random blood glucose level [13, 14], blood pressure on admission [18], cardiac ejection fraction [18], time to thrombolysis [14], patient age [13, 15], ASPECT Score on admission [15], hyperdense MCA sign [19, 20], having an anterior circulation stroke [18] and past history of stroke / CHADS score [18]. Some of these associations may be attributable to the variation in samples sizes and differences in baseline demographic and clinical characteristics (e.g., past history of co-morbidities) of individual cohorts. Time to admission was associated with functional independence at 3 months in the unadjusted analysis in our cohort, but this became insignificant in the adjusted analysis probably due to the overall delays in getting admitted or treated compared to other studies. This should not be erroneously interpreted as time to admission not having an impact on post-thrombolysis functional outcome. The overall variation in predictors across studies from different countries suggest that aiming to develop a universal system to predict post-thrombolysis functional improvements may be less relevant, and instead clinicians should focus on establishing locally adapted, evidence-based approaches to improve outcomes of their patients.

As for complications, the percentage suffering from post-thrombolysis sICHs were relatively higher in our cohort (supplementary Fig. 3) and the same was true for all cause mortality at 3 months [10, 21] (supplementary Fig. 4). Previous studies have found several associations for post-thrombolysis sICH including age, current or historical antiplatelet /anticoagulant use [18, 22, 23], serum triglyceride and fibrinogen level [18, 24], statin use [25, 26], hypertension or hyperglycaemia on admission [18], NIHSS on admission [18], anterior circulation stroke [18], leukoaraiosis on CT [17], ASPECT score and comorbidities such as atrial fibrillation [18], renal impairment [27], heart failure [18] or a CHADS2 score > 2 [18]. Some of these associations have been confirmed in a meta-analysis [28]. Interestingly, we could not replicate these observations in this study probably due to the low event rate.

Whether low dose alteplase is comparable to standard dose in efficacy and safety in Asian patients remains controversial [22]. Taiwan Thrombolytic Therapy for Acute Ischemic Stroke (TTT-AIS) study, one of the first studies to study this, showed that complications were higher (sICH, deaths and dependence at 3 months) in elderly patients receiving a dose of 0.9 mg/kg [22]. A second larger study in China which tested a range of doses from 0.6 – 0.9 mg/kg suggested that lower doses were associated with better outcomes in older patients [29]. These observations led to Japan approving a lower than standard dose of rtPA for thrombolysis based on trial data [30]. Finally, a larger international study (ENCHANTED) which recruited patients from 13 countries including populations from Europe, Asia, South America, and Australia demonstrated that low dose rtPA is not inferior to standard dose of 0.9 mg/kg [31]. However, there were little or no representation of South Asian populations in these studies. The use of low doses of rtPA was proposed in some countries due racial and genetic differences related to the functionality of fibrinogen and coagulation factors which potentially increased the risk of intracerebral haemorrhage [32]. Our observations add to the very limited evidence base in this regard from South Asia to suggest that low dose alteplase may be as effective as standard dose, but did not necessarily had a better safety profile with regard to deaths and sICH, as expected. Supplementary Table 3 illustrates studies comparing low dose vs standard dose which mostly shows no difference for efficacy outcomes. A previous meta-analysis had concluded that there is no association between alteplase dose and favourable outcome and mortality, but that the low dose may be associated with a lower incidence of sICH [33]. From a cost perspective, alteplase used in Sri Lanka is made in India and a 20 mg vial costs USD 355 while a 50 mg vial costs USD 692. If the low dose is as effective as the standard dose this could be considerably cost saving in a resource limited setting.

This study has several limitations; firstly, we recruited from a single centre which affects the generalisability of studies to the whole of Sri Lanka. Yet NHSL-c is one of the few centres where thrombolysis is available in the country and all other centres where thrombolysis is available also do have similar facilities and are supervised consultant neurologists. Secondly, Sri Lankan data may not be generalisable to other countries but as shown above in the discussion, the heterogeneity of associations for outcomes and complications highlight the need for locally relevant datasets than extrapolating findings from other countries. Thirdly, the sample size is small despite recruiting all eligible patients from all neurology units in the largest hospital in Sri Lanka located in the most populous district of the country over a 24-month period. This highlights a greater problem in gaining access to services within the critical time window for thrombolysis which may be influenced by patient-dependent (e.g., unawareness) and -independent factors (e.g., lack of infrastructure for faster transport of critically ill patients). Exploring reasons for this was beyond the scope of this study. Finally, comparison of low dose vs. standard dose alteplase should ideally be done as a randomised blinded controlled trial for conclusive results.

## Conclusion

This single centre prospective observational study of patients undergoing thrombolysis, the first of its kind from Sri Lanka, a low-middle income country, shows that NIHSS on admission to be independently associated with functional independence at 3 months post-thrombolysis. Notably the time to thrombolysis in this cohort was higher compared to similar studies in other countries which is a concern as it may indicate problems with accessing services within the critical time window. Low dose rtPA may be as good as standard dose in terms of efficacy and complications (death or sICH) for Sri Lankan patients, but this needs to be confirmed in a randomised controlled trial.

## Supplementary Information

**Additional file 1: Supplementary Table 1**. Definitions of data items used in this paper. **Supplementary Table 2**. Descriptions of prospective observational / interventional studies on thrombolysis which had assessed similar outcomes and complications as this study. **Supplementary Table 3**. A summary of previous studies that have compared low dose and stander dose of alteplase for thrombolysis.

**Additional file 2: Supplementary Figure 1**. Comparison of prospective studies on thrombolysis from literature with regard to time window from symptom onset to thrombolysis. X axis - time in minutes, Y axis – Study. Legends – Black colour = current study, light grey colour = other studies. For more details on each trial including country and dose of alteplase used, refer to supplementary Tables 2 and 3.

**Additional file 3: Supplementary Figure 2**. Comparison of prospective studies on thrombolysis from literature with regard to functional improvement at 3 months post-thrombolysis. X axis - % of patients showing a mRS of 0 or 1. Y axis – Study. Legends – Black colour = current study, light grey colour = other studies. For more details on each trial including country and dose of alteplase used, please refer to supplementary Tables 2 and 3.

**Additional file 4: Supplementary Figure 3**. Comparison of prospective studies on thrombolysis from literature with regard to symptomatic intracranial haemorrhages (sICH) following thrombolysis. X axis - % of patients experiencing sICH within 3 months of thrombolysis. Y axis – Study. Legends – Black colour = current study, light grey colour = other studies. For more details on each trial including country and dose of alteplase used, please refer to supplementary Tables 2 and 3.

**Additional file 5: Supplementary Figure 4**. Comparison of prospective studies on thrombolysis from literature with regard to 3-month all-cause mortality. X axis - % of patients who did not survive at 3 months post-thrombolysis, Y axis – Study. Legends – Black colour = current study, light grey colour = other studies. For more details on each trial including country and dose of alteplase used, please refer to supplementary Tables 2 and 3.

## Data Availability

The data set for this publication is available upon request from the authors.

## Abbreviations

DALYs: Disability adjusted life years lost
rtPA: Recombinant tissue plasminogen activator
NHSL-C: National Hospital of Sri Lanka in Colombo
AHA/ASA: American Heart Association/American Stroke Association
NIHSS: National Institutes of Health Stroke Score
NINDS: National Institute of Neurological Disorders and Stroke
mRS: modified Rankin Scale
ASPECTS: Alberta Stroke Programme early CT score
sICH: Symptomatic intracranial heamorrhages
MCA: Middle cerebral artery
