# Supplementary material for: Outcomes of stroke patients undergoing thrombolysis in Sri Lanka; an observational prospective study from a low-middle income country
Source: BMC Neurol. 2021 Nov 9;21:434. doi: 10.1186/s12883-021-02475-3 (PMC8576930; doi:10.1186/s12883-021-02475-3)
Supplement: Supplementary file 1 — Additional file 1: Supplementary Table 1. Definitions of data items used in this paper. Supplementary Table 2. Descriptions of prospective observational / interventional studies on thrombolysis which had assessed similar outcomes and complications as this study. Supplementary Table 3. A summary of previous studies that have compared low dose and stander dose of alteplase for thrombolysis. [file 12883_2021_2475_MOESM1_ESM.docx]

Supplementary table 1. Data items and definitions used in this study

| Variable | Comments |
| --- | --- |
| Age | In years |
| Body Mass index |  |
| Diabetes | Previously diagnosed and mentioned in medical records |
| Hypertension | Previously diagnosed and mentioned in medical records |
| Previous stroke | Documented history of ischemic stroke in medical records |
| Ischemic heart disease | A diagnosis of stable /unstable angina, non ST elevation or ST elevation myocardial infarction from medical records |
| Congestive cardiac failure | Clinical diagnosis of heart failure by Modified Framingham clinical criteria and confirmed by echocardiography as either heart failure with preserved ejection fraction or heart failure with low ejection fraction |
| Smoking | Non-smoker and former smoker vs current smoker (within the preceding 12 months) |
| Alcohol use | Abstainers and users with < 90 g of alcohol per week vs others (with the preceding 12-month period) |
| Regular aspirin use | Users vs non users; regular – missing no or only one dose in the preceding 2 weeks |
| Regular clopidogrel use | Users vs non users; regular – missing no or only one dose in the preceding 2 weeks |
| Regular statin use | Users vs non users; regular – missing no or only one dose in the preceding 2 weeks |
| Regular anticoagulation use | Users vs non users; regular – missing no or only one dose in the preceding 2 weeks |
| Time to admission | Time from onset of symptoms to the time stamped on the admission document in the hospital |
| Time to reperfusion | Time from onset of symptoms to starting of rtPA |
| Modified Rankin scale(1) | Assessed on admission pre-thrombolysis and 3 months after thrombolysis( mRS runs from 0-6, 0 indicates no symptoms , 5 indicates severe disability and 6 indicates death. ) (dichotomized as independent and dependent: 0-2 or 3-5) |
| National Institutes of Health – Stroke Score(2) | Assessed on admission pre-thrombolysis, 24 hours after and 3 months after thrombolysis, NIHSS is a scale with 42 points that quantify neurological deficit in 11 categories. (dichotomized as favourable and non-favourable: 0-1 or > 1) |
| Type of stroke | Anterior vs posterior circulation stroke |
| Systolic blood pressure | Blood pressure recording on admission |
| Diastolic blood pressure | Blood pressure recording on admission |
| Platelet count | Classified in to three categories based on the platelet count on admission; < 150,000, 150,000 – 300,000, >300,000/µl |
| On admission hematocrit level | Classified in to three categories as “low”, “normal” or “high”; The normal hematocrit for men is 40 - 54%; for women it is 36 - 48%. |
| Serum glucose level | Random blood glucose level on admission |
| Estimated glomerular filtration rate (eGFR) | Estimated from pre-thrombolysis serum creatinine level using the MDRD equation |
| CT - ASPECT score(3) | Is a 10-point quantitative topographic CT scan score used for patients with middle cerebral artery stroke and has also been adjusted for the posterior circulation |
| CT - Leukoariaosis(4) | Neuroimaging abnormalities (bilateral and either patchy or diffuse areas of hypodensity on CT or hyperintensity on T2-weighted MRI ) of the white matter of the brain. |
| CT - hyperdense MCA sign(5) | Focal hyperdensity of the middle cerebral artery on non-contrast brain CT and is the direct visualisation of thromboembolic material within the lumen. |

**Supplementary table 2.** Descriptions of prospective observational / interventional studies on thrombolysis which had assessed similar outcomes and complications as this study

| Publication | Year | Country | Dose (mg/kg) and time interval(minutes) | Mean age in years* | Sample size | Base line NIHSS* | Onset to treatment (Minutes) * | Favourable clinical outcome at 3 months  BI % ^Ω^ | Favourable clinical outcome at 3 months  mRS % ^Ω^ | Favourable clinical outcome at 3 months  NIHSS % ^Ω^ | sICH (%) | Mortality(%) |
| --- | --- | --- | --- | --- | --- | --- | --- | --- | --- | --- | --- | --- |
| NINDS part 1(6) | 1995 | USA | 0.9 /180 | 67±10 | 291 | 14(1-37)** | 119.7 | 54 | 47 | 38 | 6 | NA |
| NINDS part 2(6) | 1995 | USA | 0.9 /180 | 69±12 | 333 | 14 | 119.7 | 50 | 39 | 31 | 7 | 17 |
| ECASS(7) | 1995 | European countries | 1.1/360 | 69±12 | 109 | 12 | 258 | NA | 35.7 | NA | 19.8 | 17.9 |
| ECASS II)(8) | 1998 | Europe, Australia, and New Zealand | 0.9/180 | 68 | 165 | 11 | NA | NA | 42 | NA | 11.8 | 10.5 |
| ATLANTIS (9) | 2002 | America | 0.9/180 | 66 | 61 | 12 | 161 | 65.2 | 61.6 | 60.9 | 13 | 17.4 |
| J-ACT(10) | 2006 | japan | 0.6/180 | 70.9±9.8 | 103 | 15(5-30)** | 150.5 | 48.5 | 36.9 | NA | 5.8 | 9.7 |
| Suwanwela et al (11) | 2006 | Thailand | 0.9/270 | 65.5 ± 12 | 34 | 11±6.5 | 137.7 | NA | NA | NA | 5.9 | 5.9 |
| Padma MV et al(12) | 2007 | India | 0.9/270 | 66 | 54 | 14 ± 2 | 170.8 | NA | NA | NA | 0 | 1.9 |
| SITS-MOST (13) | 2007 | International | 0.9/180 | 68 | 6483 | 12 | 140 | NA | 54.8 | NA | 7.3 | 11.3 |
| Sharma SR, et al(14) | 2008 | India | 0.9/270 | 66 | 32 | 15.5 ± 2 | 150 | NA | NA | NA | 0 | 3.1 |
| Hsu YC et al(15) | 2009 | Taiwan | 0.9/270 | 63 | 43 | 18 | 134 | NA | 33 | NA | 4.7 | 9.3 |
| Toyoda K et al(16) | 2009 | Japan | 0.6/180 | 72±12 | 600 | 13 (7.3–19)** | 145 | NA | 33.2 | NA | 3.8 | 7.2 |
| Wasay (17) | 2010 | Pakistan | 0.9/270 | 62 | 21 | NA | 169 | NA | NA | NA | 24 | 19 |
| Sharma VK et al(18) | 2010 | Singapore | 0.9/270 | 62±13 | 82 | 15 | 155 | NA | 59 | NA | 1.2 | 13 |
| Sharma VK et al(18) | 2010 | Singapore | 0.67 /270 | 55±12 | 48 | 12 | 165 | NA | 35 | NA | 14.6 | 10 |
| J-ACT II  (19) | 2010 | Japan | 0.6/180 | 70.3±11.5 | 58 | 12(5-22)** | 132 | NA | 46.6 | NA | 0 | 1.7 |
| Nguyen TH et al(20) | 2010 | Vietnam | 0.9/180 | 58 ± 14 | 73 | 12(15-23)** | 145±33 | NA | 34.2 | NA | 5.5 | 12.5 |
| Nguyen TH et al(20) | 2010 | Vietnam | 0.62/180 | 57±13 | 48 | 12(15-23)** | 141±33 | NA | 56.3 | NA | 2.1 | 2.1 |
| TTT-AIS  (21) | 2010 | Taiwan | 0.90/180 | 64.9±11.7 | 125 | 15.9±5.6 | 137.5±39.4 | NA | 48.7^@^ | NA | 8.0 | 12.8 |
| TTT-AIS  (21) | 2010 | Taiwan | 0.72/180 | 66.7±13.3 | 116 | 14.9±6.0 | 141.6±34.9 | NA | 58.8^@^ | NA | 2.6 | 6.9 |
| Boddu et al(22) | 2010 | India | 0.9/270 | 53 ± 12.3 | 72 | 10±6 | 152 | NA | 41.5 | NA | 4.1 | 9 |
| Zhou XY et al (23) | 2010 | China | 0.6–0.7/180 | 69.8 ± 8.6 | 23 | 12.6 ± 6.8 | 170.3 ± 43.9 | NA | 34.8 | NA | 4.3 | 17.4 |
| Zhou XY et al (23) | 2010 | China | 0.8/180 | 72.9 ± 8.7 | 31 | 12.7 ± 5.0 | 74.3 ± 45.2 | NA | 38.7 | NA | 3.2 | 16.1 |
| Zhou XY et al (23) | 2010 | China | 0.9/180 | 72.7 ± 10.7 | 51 | 13.0 ± 6.3 | 153.5 ± 53.0 | NA | 51 | NA | 3.9 | 11.8 |
| J-MARS (24) | 2010 | Japan | 0.6/180 | 72 | 7492 | 15 | 133 | NA | 33.1 | NA | 4.4 | 13.1 |
| Muengtaweepongsa S et al(25) | 2012 | Thailand | 0.9/180 | 65 | 100 | 15 | 160 | NA | 42 | NA | 2 | 14 |
| Chen CH et al(26) | 2012 | Taiwan | 0.9/180 | 67.9 | 156 | 13.1 | 141 | NA | 38.4 | NA | 1.3 | 5.8 |
| Chen CH et al(26) | 2012 | Taiwan | 0.7/180 | 67.9 | 105 | 13.3 | 144 | NA | 41.1 | NA | 3.8 | 7.6 |
| Salam KA et al (27) | 2014 | Inda | 0.9/270 | 62 | 31 | 10(5-22)** | 65 | NA | 48^@^ | NA | NA | 6.5 |
| Kim BJ et al(28) | 2015 | South Korea | 0.9/270 | 68.2±12.3 | 1076 | 10.5±6.0 | 126 | NA | 35.3 | NA | 6.4 | 14.0 |
| Kim BJ et al(28) | 2015 | South Korea | 0.6/270 | 69.0±12.7 | 450 | 13.9±7.0 | 126 | NA | 32.4 | NA | 8.4 | 12.7 |
| Enchanted (29) | 2016 | International | 0.9/270 | 67 | 1643 | 8 | 170 | NA | NA | NA | 8 | 10.3 |
| Enchanted (29) | 2016 | International | 0.6/270 | 68 | 1654 | 8 | 170 | NA | NA | NA | 5.9 | 8.5 |
| Zhao G et al(30) | 2018 | China | 0.9/270 | 64 | 371 | 13 | 215 | NA | 37.5 | NA | 5.9 | 7.3 |
| Zhao G et al(30) | 2018 | China | 0.6–0.89 /270 | 65 | 1115 | 13 | 211 | NA | 36.1 | NA | 2.2 | 5.5 |
| Mai DT et al(31) | 2021 | Vietnam | 0.9/270 | 63.6±11.8 | 34 | 14 | 157.1±43.6 | NA | 64.8^@^ | NA | 11.8 | 8.8 |
| Mai DT et al(31) | 2021 | Vietnam | 0.6/270 | 62.4±11.4 | 73 | 16 | 175.6±43.3 | NA | 68.5^@^ | NA | 5.5 | 2.7 |
| Current study | 2021 | Sri lanka | 0.7-0.9/270 | 60 ± 12.17 | 89 | 13 ± 5.21 | 174 ± 56.50 | 72.5 ^$^ | 46.3 | 33.3% | 21.3 | 11.3 |

* = Expressed as mean or mean ±SD unless otherwise specified. ** = Expressed as mean (Range of data)

mRS = modified Rankin scale, BI = Barthel index, NIHSS = National Institutes of Health stroke scale

Ω = % of patients with a favourable response according to different scoring systems. Scores of 95 or 100 on the BI, <1 on the NIHSS and the mRS were considered as a favourable outcome.

@= mRS <2 was considered as favourable response in these studies.

$ = BI > 60 was considered as favourable response in these studies

Supplementary table 3. A summary of previous studies that have compared low dose and stander dose of alteplase for thrombolysis.

| Publication | Year | country | Dose ( mg/Kg) | Efficacy and functional outcome* | Incidence of sICH or/mortality * | Comments/Conclusion |
| --- | --- | --- | --- | --- | --- | --- |
| (10) | 2006 | Japan | LD = 0.6, SD = already published 0.9 | No difference | No difference | LD in Japan had similar clinical efficacy and safety compared to SD in North America and the European Union |
| (16) | 2009 | Japan | LD = 0.6, SD = already published 0.9 | No difference | No difference | LD had similar outcomes in three-month outcomes compared to post marketing surveys using 0.9 mg/kg alteplase |
| (21) | 2010 | Taiwan – Chinese | SD = 0.90 ± 0.02  LD = 0.72 ± 0.07 | Favours LD | Favours LD | LD has better outcomes and safety compared to SD of alteplase in Chinese patients |
| (19) | 2010 | Japan | LD = 0.6, SD = already published 0.9 | No difference | No difference | The rates of recanalization and favourable outcome is similar to that reported SD outcomes. |
| (24) | 2010 | Japan | LD = 0.6, SD = already published 0.9 | No difference | No difference | LD within 3 hours is safe and effective similar to already published data with SD Japanese. |
| (20) | 2010 | Vietnam | SD = 0.9  LD 0.62 (0.6-0.86) | Favours LD | Favours LD | LD is safe and effective in the treatment compared to SD in selected Vietnamese population. |
| (18) | 2010 | Singapore | SD= 0.9  LD = 0.67(0.50.71) | Favours SD | Favours SD | SD was effective and safe compared to LD in multi-ethnic Asian population in Singapore |
| (26) | 2012 | Taiwan | SD = 0.9  LD = 0.7 | No difference | No difference | SD for acute ischemic stroke in an Asian population has similar efficacy and safety compared to LD |
| (28) | 2015 | South Korea | SD = 0.9  LD = 0.6 | No difference | No difference | LD has similar effectiveness and safety compared to the SD . |
| (29) | 2016 | International | SD = 0.9  LD = 0.6 | No difference | Favours LD | LD was not noninferior to SD with respect to death and disability. Significantly fewer sICH with LD. |
| (32) | 2017 | International | SD = 0.9  LD = 0.6 | No difference | No difference | LD was not superior to the effects of SD on death or disability. |
| (33) | 2017 | International | SD = 0.9  LD = 0.6 | Favours LD | No difference | LD alteplase may improve outcomes in patients on prior antiplatelet. |
| (30) | 2018 | China | SD = 0.9  LD = 0.6 | No difference | Favours LD | The efficacy of LD is similar to the SD, but with higher safety |
| (31) | 2021 | Vietnam | SD = 0.9  LD = 0.6 | No difference | No difference | LD before thrombectomy had similar clinical outcomes compared SD |
| (34) | 2021 | Egypt | SD = 0.9  LD = 0.6 | No difference | No difference | No statistical significant difference between LD and SD with regards to safely and outcome. Fewer sICH in LD group. |

Reference

1. Rankin J. Cerebral vascular accidents in patients over the age of 60. II. Prognosis. Scott Med J. 1957;2(5):200-15.

2. Lyden P, Brott T, Tilley B, Welch KM, Mascha EJ, Levine S, et al. Improved reliability of the NIH Stroke Scale using video training. NINDS TPA Stroke Study Group. Stroke. 1994;25(11):2220-6.

3. Barber PA, Demchuk AM, Zhang J, Buchan AM. Validity and reliability of a quantitative computed tomography score in predicting outcome of hyperacute stroke before thrombolytic therapy. ASPECTS Study Group. Alberta Stroke Programme Early CT Score. Lancet. 2000;355(9216):1670-4.

4. Pantoni L, Garcia JH. Pathogenesis of leukoaraiosis: a review. Stroke. 1997;28(3):652-9.

5. Tomsick T, Brott T, Barsan W, Broderick J, Haley EC, Spilker J, et al. Prognostic value of the hyperdense middle cerebral artery sign and stroke scale score before ultraearly thrombolytic therapy. AJNR Am J Neuroradiol. 1996;17(1):79-85.

6. National Institute of Neurological D, Stroke rt PASSG. Tissue plasminogen activator for acute ischemic stroke. N Engl J Med. 1995;333(24):1581-7.

7. Hacke W, Kaste M, Fieschi C, Toni D, Lesaffre E, von Kummer R, et al. Intravenous thrombolysis with recombinant tissue plasminogen activator for acute hemispheric stroke. The European Cooperative Acute Stroke Study (ECASS). JAMA. 1995;274(13):1017-25.

8. Hacke W, Kaste M, Fieschi C, von Kummer R, Davalos A, Meier D, et al. Randomised double-blind placebo-controlled trial of thrombolytic therapy with intravenous alteplase in acute ischaemic stroke (ECASS II). Second European-Australasian Acute Stroke Study Investigators. Lancet. 1998;352(9136):1245-51.

9. Albers GW, Clark WM, Madden KP, Hamilton SA. ATLANTIS trial: results for patients treated within 3 hours of stroke onset. Alteplase Thrombolysis for Acute Noninterventional Therapy in Ischemic Stroke. Stroke. 2002;33(2):493-5.

10. Yamaguchi T, Mori E, Minematsu K, Nakagawara J, Hashi K, Saito I, et al. Alteplase at 0.6 mg/kg for acute ischemic stroke within 3 hours of onset: Japan Alteplase Clinical Trial (J-ACT). Stroke. 2006;37(7):1810-5.

11. Suwanwela NC, Phanthumchinda K, Likitjaroen Y. Thrombolytic therapy in acute ischemic stroke in Asia: The first prospective evaluation. Clin Neurol Neurosurg. 2006;108(6):549-52.

12. Padma MV, Singh MB, Bhatia R, Srivastava A, Tripathi M, Shukla G, et al. Hyperacute thrombolysis with IV rtPA of acute ischemic stroke: efficacy and safety profile of 54 patients at a tertiary referral center in a developing country. Neurol India. 2007;55(1):46-9.

13. Wahlgren N, Ahmed N, Davalos A, Ford GA, Grond M, Hacke W, et al. Thrombolysis with alteplase for acute ischaemic stroke in the Safe Implementation of Thrombolysis in Stroke-Monitoring Study (SITS-MOST): an observational study. Lancet. 2007;369(9558):275-82.

14. Sharma SR, Sharma N. Hyperacute thrombolysis with recombinant tissue plasminogen activator of acute ischemic stroke: feasibility and effectivity from an Indian perspective. Ann Indian Acad Neurol. 2008;11(4):221-4.

15. Hsu YC, Sung SF, Ong CT, Wu CS, Su YH. Intravenous thrombolytic therapy for acute ischemic stroke: the experience of a community hospital. Acta Neurol Taiwan. 2009;18(1):14-20.

16. Toyoda K, Koga M, Naganuma M, Shiokawa Y, Nakagawara J, Furui E, et al. Routine use of intravenous low-dose recombinant tissue plasminogen activator in Japanese patients: general outcomes and prognostic factors from the SAMURAI register. Stroke. 2009;40(11):3591-5.

17. Wasay M, Barohi H, Malik A, Yousuf A, Awan S, Kamal AK. Utilization and outcome of thrombolytic therapy for acute stroke in Pakistan. Neurol Sci. 2010;31(2):223-5.

18. Sharma VK, Tsivgoulis G, Tan JH, Wong LY, Ong BK, Chan BP, et al. Feasibility and safety of intravenous thrombolysis in multiethnic Asian stroke patients in Singapore. J Stroke Cerebrovasc Dis. 2010;19(6):424-30.

19. Mori E, Minematsu K, Nakagawara J, Yamaguchi T, Sasaki M, Hirano T, et al. Effects of 0.6 mg/kg intravenous alteplase on vascular and clinical outcomes in middle cerebral artery occlusion: Japan Alteplase Clinical Trial II (J-ACT II). Stroke. 2010;41(3):461-5.

20. Nguyen TH, Truong AL, Ngo MB, Bui CT, Dinh QV, Doan TC, et al. Patients with thrombolysed stroke in Vietnam have an excellent outcome: results from the Vietnam Thrombolysis Registry. Eur J Neurol. 2010;17(9):1188-92.

21. Chao AC, Hsu HY, Chung CP, Liu CH, Chen CH, Teng MM, et al. Outcomes of thrombolytic therapy for acute ischemic stroke in Chinese patients: the Taiwan Thrombolytic Therapy for Acute Ischemic Stroke (TTT-AIS) study. Stroke. 2010;41(5):885-90.

22. Boddu DB, Srinivasarao Bandaru VC, Reddy PG, Madhusudan M, Rukmini MK, Suryaprabha T, et al. Predictors of major neurological improvement after intravenous thrombolysis in acute ischemic stroke: a hospital-based study from south India. Neurol India. 2010;58(3):403-6.

23. Zhou XY, Wang SS, Collins ML, Davis SM, Yan B. Efficacy and safety of different doses of intravenous tissue plasminogen activator in Chinese patients with ischemic stroke. J Clin Neurosci. 2010;17(8):988-92.

24. Nakagawara J, Minematsu K, Okada Y, Tanahashi N, Nagahiro S, Mori E, et al. Thrombolysis with 0.6 mg/kg intravenous alteplase for acute ischemic stroke in routine clinical practice: the Japan post-Marketing Alteplase Registration Study (J-MARS). Stroke. 2010;41(9):1984-9.

25. Muengtaweepongsa S, Dharmasaroja P, Kummark U. Outcomes of intravenous thrombolytic therapy for acute ischemic stroke with an integrated acute stroke referral network: initial experience of a community-based hospital in a developing country. J Stroke Cerebrovasc Dis. 2012;21(1):42-6.

26. Chen CH, Hsieh CY, Lai TB, Chuang MT, Chen WL, Sun MC. Optimal dose for stroke thrombolysis in Asians: low dose may have similar safety and efficacy as standard dose. J Thromb Haemost. 2012;10(7):1270-5.

27. Salam KA, Ummer K, Pradeep Kumar VG, Noone ML. Intravenous thrombolysis for acute ischemic stroke in the 3- to 4.5-hour window--the Malabar experience. Int J Stroke. 2014;9(4):426-8.

28. Kim BJ, Han MK, Park TH, Park SS, Lee KB, Lee BC, et al. Low-Versus Standard-Dose Alteplase for Ischemic Strokes Within 4.5 Hours: A Comparative Effectiveness and Safety Study. Stroke. 2015;46(9):2541-8.

29. Anderson CS, Robinson T, Lindley RI, Arima H, Lavados PM, Lee TH, et al. Low-Dose versus Standard-Dose Intravenous Alteplase in Acute Ischemic Stroke. N Engl J Med. 2016;374(24):2313-23.

30. Zhao G, Huang T, Zheng M, Cui Y, Liu Y, Cheng Z, et al. Comparative Analysis on Low- and Standard-Dose Regimes of Alteplase Thrombolytic Therapy for Acute Ischemic Stroke: Efficacy and Safety. Eur Neurol. 2018;79(1-2):68-73.

31. Mai DT, Dao VP, Nguyen VC, Vu DL, Nguyen TD, Vuong XT, et al. Low-Dose vs. Standard-Dose Intravenous Alteplase in Bridging Therapy Among Patients With Acute Ischemic Stroke: Experience From a Stroke Center in Vietnam. Front Neurol. 2021;12:653820.

32. Wang X, Robinson TG, Lee TH, Li Q, Arima H, Bath PM, et al. Low-Dose vs Standard-Dose Alteplase for Patients With Acute Ischemic Stroke: Secondary Analysis of the ENCHANTED Randomized Clinical Trial. JAMA Neurol. 2017;74(11):1328-35.

33. Robinson TG, Wang X, Arima H, Bath PM, Billot L, Broderick JP, et al. Low- Versus Standard-Dose Alteplase in Patients on Prior Antiplatelet Therapy: The ENCHANTED Trial (Enhanced Control of Hypertension and Thrombolysis Stroke Study). Stroke. 2017;48(7):1877-83.

34. Salem GM, El-Sheik WM, El-Shanawany BG, Afifi KH. Low versus standard dose intravenous alteplase in the treatment of acute ischemic stroke in Egyptian patients. Neurosciences (Riyadh). 2021;26(2):179-85.
