# Supplementary figures and images for: Outcomes of stroke patients undergoing thrombolysis in Sri Lanka; an observational prospective study from a low-middle income country
Source: BMC Neurol. 2021 Nov 9;21:434. doi: 10.1186/s12883-021-02475-3 (PMC8576930; doi:10.1186/s12883-021-02475-3)

STUDY

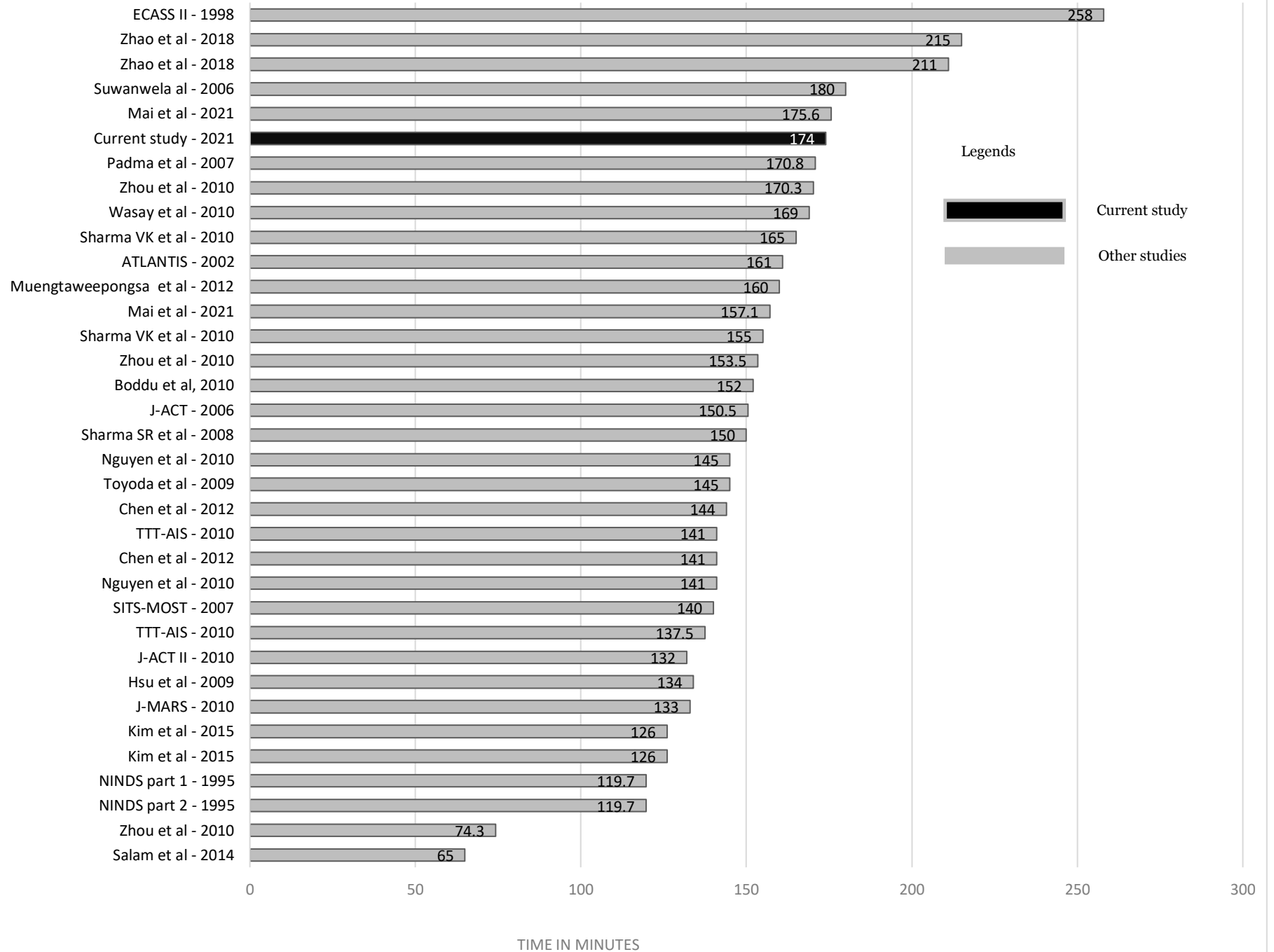

Supplement: Supplementary file 2 — Additional file 2: Supplementary Figure 1. Comparison of prospective studies on thrombolysis from literature with regard to time window from symptom onset to thrombolysis. X axis - time in minutes, Y axis – Study. Legends – Black colour = current study, light grey colour = other studies. For more details on each trial including country and dose of alteplase used, refer to supplementary Tables 2 and 3. [file 12883_2021_2475_MOESM2_ESM.pdf]

STUDY

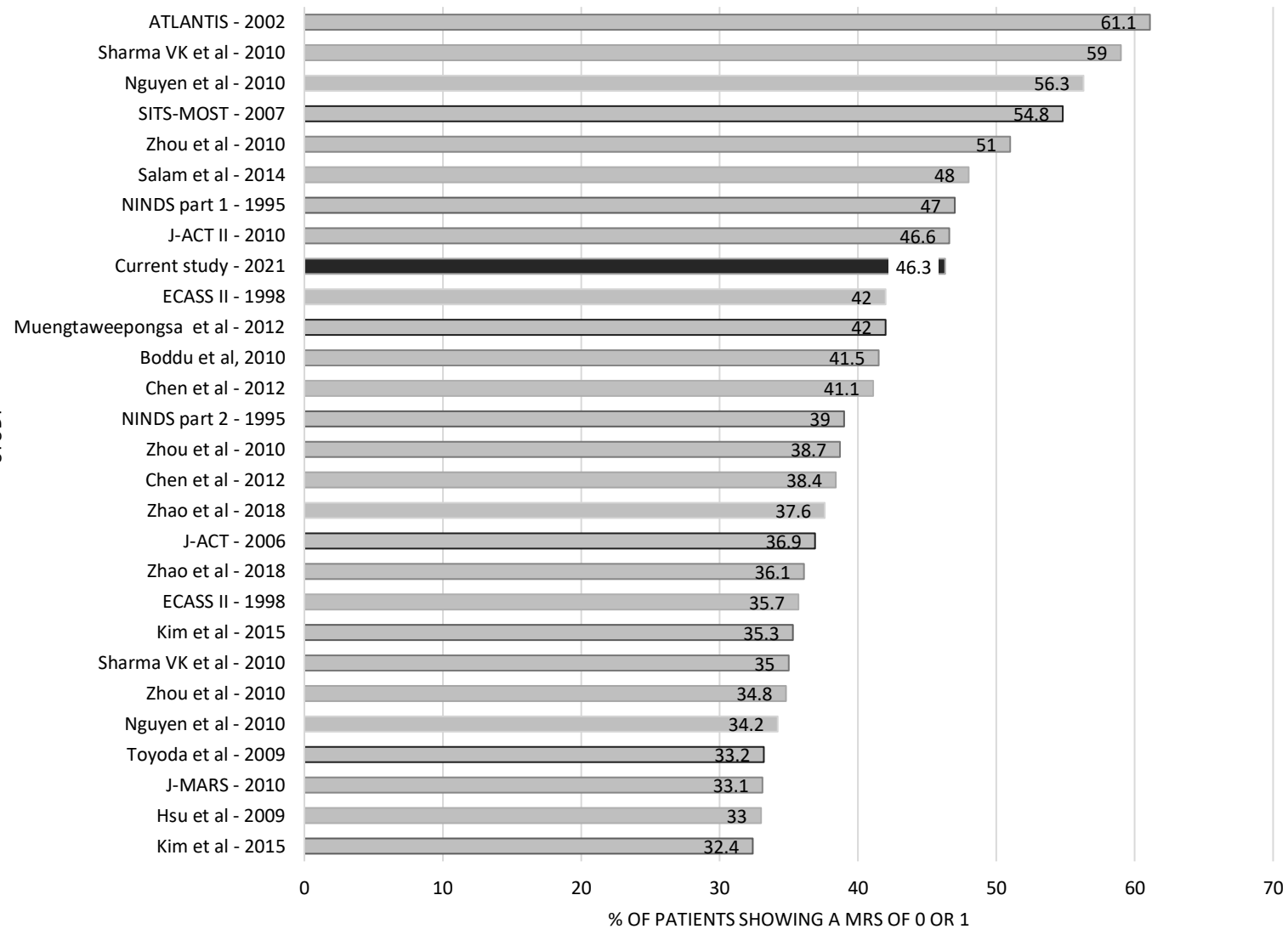

Supplement: Supplementary file 3 — Additional file 3: Supplementary Figure 2. Comparison of prospective studies on thrombolysis from literature with regard to functional improvement at 3 months post-thrombolysis. X axis - % of patients showing a mRS of 0 or 1. Y axis – Study. Legends – Black colour = current study, light grey colour = other studies. For more details on each trial including country and dose of alteplase used, please refer to supplementary Tables 2 and 3. [file 12883_2021_2475_MOESM3_ESM.pdf]

STUDY

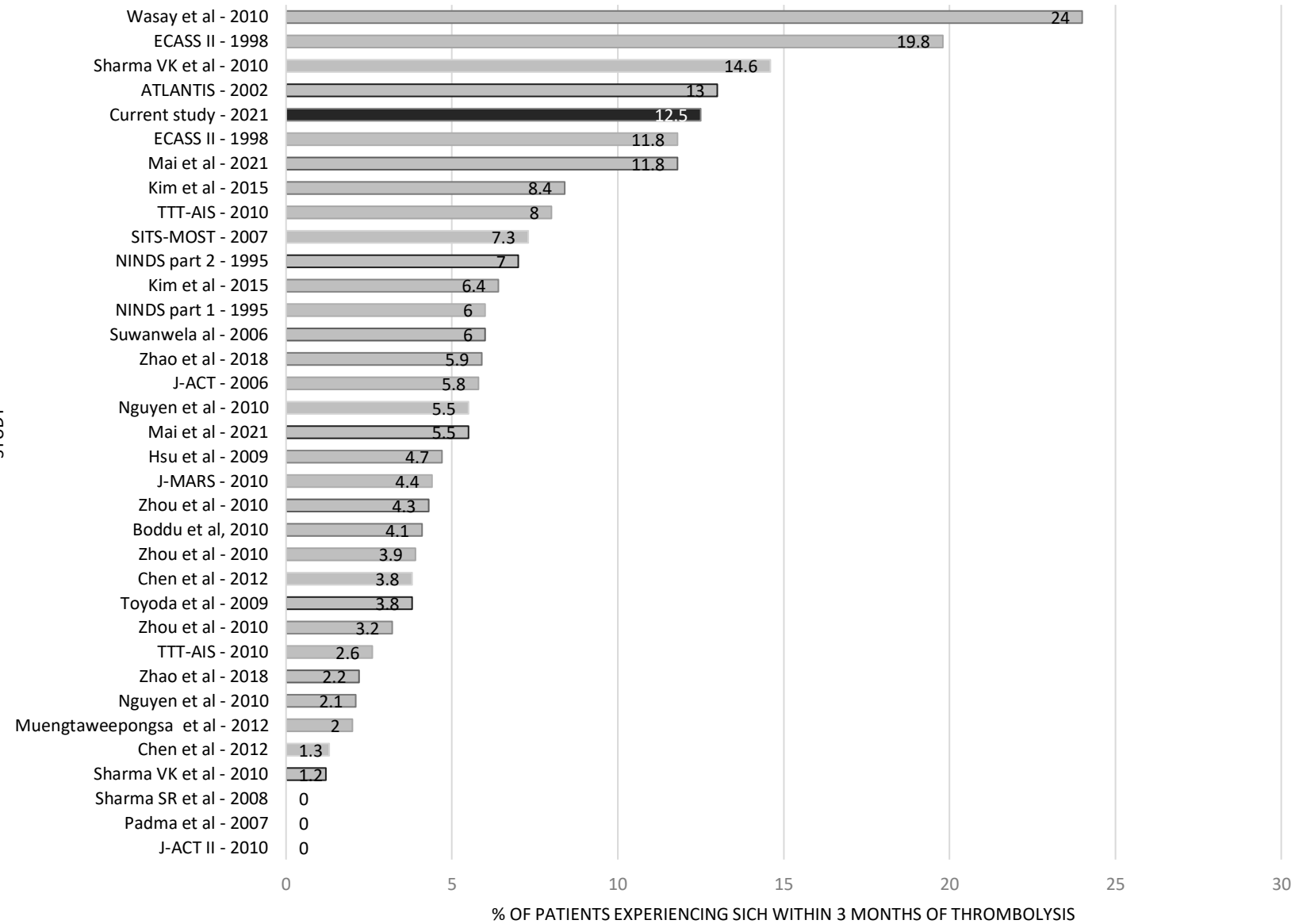

Supplement: Supplementary file 4 — Additional file 4: Supplementary Figure 3. Comparison of prospective studies on thrombolysis from literature with regard to symptomatic intracranial haemorrhages (sICH) following thrombolysis. X axis - % of patients experiencing sICH within 3 months of thrombolysis. Y axis – Study. Legends – Black colour = current study, light grey colour = other studies. For more details on each trial including country and dose of alteplase used, please refer to supplementary Tables 2 and 3. [file 12883_2021_2475_MOESM4_ESM.pdf]
