# Supplementary material for: Outcomes of stroke patients undergoing thrombolysis in Sri Lanka; an observational prospective study from a low-middle income country
Source: BMC Neurol. 2021 Nov 9;21:434. doi: 10.1186/s12883-021-02475-3 (PMC8576930; doi:10.1186/s12883-021-02475-3)
Supplement: Supplementary file 5 — Additional file 5: Supplementary Figure 4. Comparison of prospective studies on thrombolysis from literature with regard to 3-month all-cause mortality. X axis - % of patients who did not survive at 3 months post-thrombolysis, Y axis – Study. Legends – Black colour = current study, light grey colour = other studies. For more details on each trial including country and dose of alteplase used, please refer to supplementary Tables 2 and 3. [file 12883_2021_2475_MOESM5_ESM.pdf]

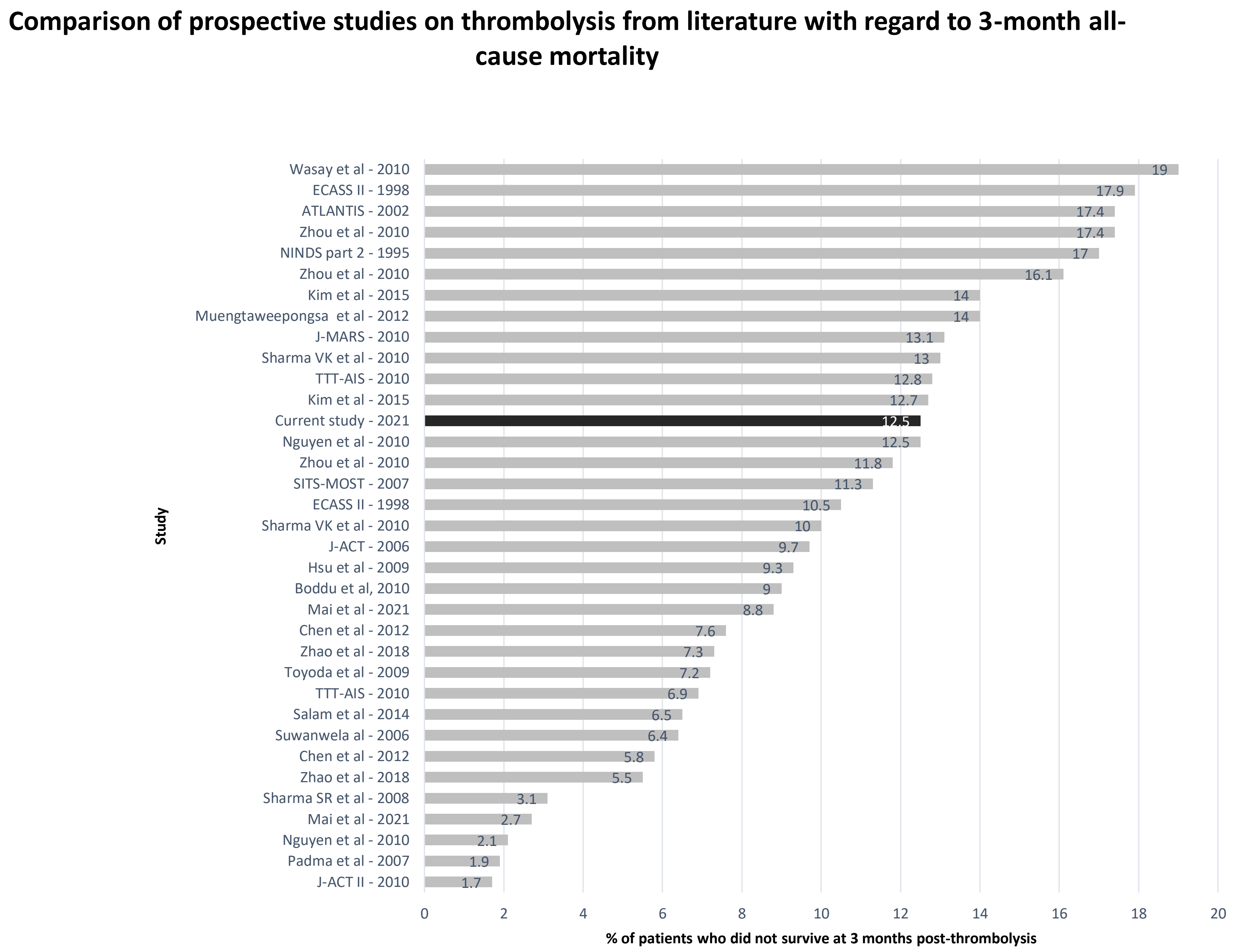
Comparison of prospective studies on thrombolysis from literature with regard to 3-month all-cause mortality
Study
Wasay et al - 2010 19
ECASS II - 1998 17.9
ATLANTIS - 2002 17.4
Zhou et al - 2010 17.4
NINDS part 2 - 1995 17
Zhou et al - 2010 16.1
Kim et al - 2015 14
Muengtaweepongsa et al - 2012 14
J-MARS - 2010 13.1
Sharma VK et al - 2010 13
TTT-AIS - 2010 12.8
Kim et al - 2015 12.7
Current study - 2021 12.5
Nguyen et al - 2010 12.5
Zhou et al - 2010 11.8
SITS-MOST - 2007 11.3
ECASS II - 1998 10.5
Sharma VK et al - 2010 10
J-ACT - 2006 9.7
Hsu et al - 2009 9.3
Boddu et al, 2010 9
Mai et al - 2021 8.8
Chen et al - 2012 7.6
Zhao et al - 2018 7.3
Toyoda et al - 2009 7.2
TTT-AIS - 2010 6.9
Salam et al - 2014 6.5
Suwanwela al - 2006 6.4
Chen et al - 2012 5.8
Zhao et al - 2018 5.5
Sharma SR et al - 2008 3.1
Mai et al - 2021 2.7
Nguyen et al - 2010 2.1
Padma et al - 2007 1.9
J-ACT II - 2010 1.7
0 2 4 6 8 10 12 14 16 18 20
% of patients who did not survive at 3 months post-thrombolysis
